# The dynamic genetic repertoire of microbial communities

**DOI:** 10.1111/j.1574-6976.2008.00144.x

**Published:** 2008-12-01

**Authors:** Paul Wilmes, Sheri L Simmons, Vincent J Denef, Jillian F Banfield

**Affiliations:** 1 Department of Earth and Planetary Science, University of California at BerkeleyBerkeley, CA, USA; 2 Department of Environmental Science, Policy, and Management, University of California at BerkeleyBerkeley, CA, USA

**Keywords:** community genomics, CRISPR, genetic heterogeneity, metagenomics, population genomics, virus–host dynamics

## Abstract

Community genomic data have revealed multiple levels of variation between and within microbial consortia. This variation includes large-scale differences in gene content between ecosystems as well as within-population sequence heterogeneity. In the present review, we focus specifically on how fine-scale variation within microbial and viral populations is apparent from community genomic data. A major unresolved question is how much of the observed variation is due to neutral vs. adaptive processes. Limited experimental data hint that some of this fine-scale variation may be in part functionally relevant, whereas sequence-based and modeling analyses suggest that much of it may be neutral. While methods for interpreting population genomic data are still in their infancy, we discuss current interpretations of existing datasets in the light of evolutionary processes and models. Finally, we highlight the importance of virus–host dynamics in generating and shaping within-population diversity.

## Introduction

Microbial ecology is undergoing a technology-driven renaissance that challenges our understanding of natural microbial communities. The application of molecular tools, from 16S rRNA gene sequencing to community genomic and postgenomic methods, has provided unprecedented insights into the genetic and physiological dynamics within complex microbial assemblages. We can now obtain an incredibly detailed view of genetic and phenotypic diversity. The novelty and depth of these data is a challenge to previous conceptual paradigms of microbial community structure and function.

While considerable genetic variation between closely related microbial strains is apparent from isolate genome-sequencing studies (e.g. Alm *et al.*, 1999; Parkhill *et al.*, 2000; Tettelin *et al.*, 2005), the extent of variation detected in natural populations with genomic techniques is far greater (e.g. Tyson *et al.*, 2004; Venter *et al.*, 2004; García-Martín *et al.*, 2006; Rusch *et al.*, 2007). Studies in multiple environments indicate that fine-scale genetic variation within populations is a hallmark of natural microbial assemblages, and that it is at least in part functionally relevant (Frias-Lopez *et al.*, 2008; Wilmes *et al.*, 2008a).

**Fig. 1:**
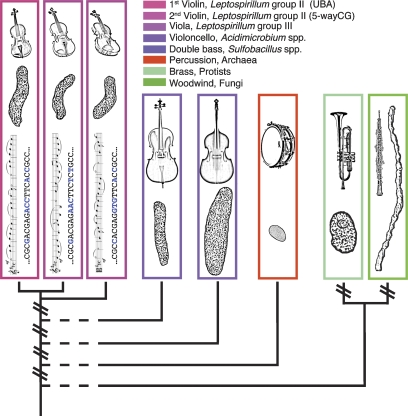
The microbial orchestra analogy showing relatedness of individual community members in acid mine drainage biofilms with corresponding instrumental groups.

To illustrate the importance of structure, variation within populations, and fine-tuning by evolutionary forces, we use the analogy of a symphony orchestra (Fig. 1). We illustrate our analogy with the example of acid mine drainage (AMD) biofilms growing within subsurface sulfuric acid solutions (pH *c*. 1, *c*. 40 °C) underground within the Richmond Mine (Iron Mountain, Redding, CA). In these biofilms, different species are partitioned into distinct ecological niches (Wilmes *et al.*, 2008b) analogous to the specific seating arrangement of different instruments. AMD biofilms are dominated by the chemoautotrophic *Nitrospira* phylum bacteria *Leptospirillum* groups II and III. *Leptospirillum* group II is the predominant member of the biofilm community and, hence, in the analogy, is associated with the violin section (Fig. 1). Its less abundant relative, *Leptosprillum* group III, is represented by the violas (Fig. 1). *Leptospirillum* group II can be broadly classified into two sequence types, 5-way CG and UBA (Tyson *et al.*, 2004; Lo *et al.*, 2007) and, hence, these are affiliated with the first and second violins, respectively (Fig. 1). Further fine-scale genetic variation within each *Leptospirillum* group II population is apparent from extensive population genomic data (Simmons *et al.*, 2008) and corresponds to the unique sound characteristics of each individual violin in the orchestra. More phylogenetically distinct organisms are equivalent to more distantly related instruments (Fig. 1).

The relative abundances and arrangement of organisms within a community may vary considerably according to environmental conditions, as do instrument numbers and seating arrangement according to performance space and composition. Just as a conductor shapes the membership, arrangement, and sound of an instrumental orchestra, natural evolutionary processes shape microbial communities. Here we present an overview of the types of population-level variations detected by community genomic (metagenomic) studies, followed by a discussion of how these data can be used to test the role of specific evolutionary processes involved in structuring communities. In particular, we focus on the importance of host–viral interactions. With the advent of functional postgenomic methodologies, i.e. transcriptomics, proteomics, and metabolomics, we are now able to listen to the ‘tunes’ played by microbial orchestras. This type of ecosystem-level analysis was recently reviewed by Raes & Bork (2008). We argue that a focus on fine-scale variation is essential to achieve a more complete understanding of microbial community function.

## The genetic repertoire of microbial communities

Recent studies based on PCR amplification and pyrosequencing of 16S rRNA gene fragments have revealed a vast phylotypic diversity in a wide range of microbial habitats [e.g. ocean (Sogin *et al.*, 2006), soil (Roesch *et al.*, 2007), and air (Tringe *et al.*, 2008)]. Although these approaches provide estimates of species richness within a given community, they are unable to resolve the true genetic diversity contained within microbial populations. Genome plasticity causes extensive variations in gene content between closely related strains of the same species (Medini *et al.*, 2005). Based on DNA reassociation kinetics of pooled genomic DNA, Gans *et al.* (2005) estimated that 1 g of pristine soil may contain 10^6^ distinct genotypes. This number far exceeds phylotypic diversity estimates for soil [e.g. 52 000 phylotypes (Roesch *et al.*, 2007)]. Consequently, due to the dynamic nature of microbial genomes, phylotypic diversity may not correlate well with genotypic and phenotypic diversity, and, hence, genotypic richness within a given sample cannot be inferred from rRNA surveys.

Community genomics (metagenomics) based on random shotgun sequencing of microbial community DNA goes far beyond marker gene surveys to provide an in-depth look at the genotypic richness within populations. The concept of sequencing genomic DNA directly from the environment was first suggested by Norman Pace (Pace *et al.*, 1985) and first implemented in the 1990s (Schmidt *et al.*, 1991; Stein *et al.*, 1996; Schleper *et al.*, 1998; Vergin *et al.*, 1998). It initially involved sequencing large inserts of DNA derived from microbial communities. Functional genes of interest were linked to community members through phylogenetically informative marker genes. A notable discovery from this approach was the presence of bacterial rhodopsin in the surface ocean (Béjà*et al.*, 2000). The high-throughput sequencing of environmental DNA was pioneered on viral communities (Breitbart *et al.*, 2002), echoing the first complete isolate genome ever sequenced, bacteriophage ΦX174 (Sanger *et al.*, 1977). Large-scale sequencing of bacterial and archaeal communities followed shortly thereafter (e.g. Schmeisser *et al.*, 2003; Tyson *et al.*, 2004; Venter *et al.*, 2004). As anticipated only 3 years ago (Allen & Banfield, 2005), microbial communities are currently being sequenced en masse. At the time of writing (June 2008) >30 metagenomic studies had been published (Table 1).

**Table 1:** Overview of random microbial community sequencing studies in chronological order

Microbiome	Organism(s) of interest	Number of bases sequenced	Sequencing technique	Reference
Seawater	Viruses	NA*	Small-insert library, Sanger sequencing	Breitbart *et al.* (2002)
Drinking water network	Bacteria	>2 Mbp	Small-insert library, Sanger sequencing	Schmeisser *et al.* (2003)
Human feces	Viruses	371 kbp	Small-insert library, Sanger sequencing	Breitbart *et al.* (2003)
Acid mine drainage	Archaea and bacteria	76.2 Mbp	Small-insert library, Sanger sequencing	Tyson *et al.* (2004)
Sargasso Sea	Archaea and bacteria	*c*. 1.625 Gbp	Small-insert library, Sanger sequencing	Venter *et al.* (2004)
Near-shore marine sediment	Viruses	NA*	Small-insert library, Sanger sequencing	Breitbart *et al.* (2004a, b)
Whale falls Farm soil	Archaea, bacteria and eukarya Archaea, bacteria and eukarya	75 Mbp 100 Mbp	Small-insert library, Sanger sequencing Small-insert library, Sanger sequencing	Tringe *et al.* (2005)Tringe *et al.* (2005)
Equine feces	Viruses	178 kbp	Small-insert library, Sanger sequencing	Cann *et al.* (2005)
Cave bear fossil	Cave bear	*c*. 2 Mbp	Small-insert library, Sanger sequencing	Noonan *et al.* (2005)
Human feces	Viruses	NA*	Small-insert library, Sanger sequencing	Zhang *et al.* (2006)
Mine water	Archaea, bacteria, eukarya, and viruses	*c*. 74 Mbp	Pyrosequencing	Edwards *et al.* (2006)
Ocean	Viruses	*c*. 181 Mbp	Pyrosequencing	Angly *et al.* (2006)
Anammox sludge bioreactor	‘*Candidatus* Kuenenia stuttgartiensis’	NA*	Medium- and large-insert library, Sanger sequencing	Strous *et al.* (2006)
North Pacific Subtropical Gyre	Archaea, bacteria, and viruses	64 Mbp	Large-insert library, Sanger sequencing	DeLong *et al.* (2006)
Mammoth fossil	Mammoth	28 Mbp	Pyrosequencing	Poinar *et al.* (2006)
Human distal gut	Archaea and bacteria	*c*. 78 Mbp	Small-insert library, Sanger sequencing	Gill *et al.* (2006)
Seawater	RNA viruses	NA*	Small-insert library, Sanger sequencing	Culley *et al.* (2006)
Phosphate removal sludges	‘*Candidatus* Accumulibacter phosphatis’	*c*. 176 Mbp	Small- and medium-insert library, Sanger sequencing	García-Martín *et al.* (2006)
*Cinara cedris*	*Buchnera aphidicola* BCcs endosymbiont	NA*	NA*	Perez-Brocal *et al.* (2006)
*Olavius algarvensis*	Gamma- and deltaproteobacterial endosymbionts	204 Mbp	Small- and large-insert library, Sanger sequencing	Woyke *et al.* (2006)
Neanderthal fossil	*Homo neanderthalensis*	NA*	Pyrosequencing	Green *et al.* (2006)
Mouse gut	Archaea, bacteria, eukarya, and viruses	199.5 Mbp	Small-insert libraries, Sanger sequencing; pyrosequencing	Turnbaugh *et al.* (2006)
Solar saltern	*Haloquadratum walsbyi*	*c*. 600 kbp	Large-insert library, Sanger sequencing and pyrosequencing	Cuadros-Orellana *et al.* (2007)
Acid mine drainage	Archaea and bacteria	*c*. 100 Mbp	Small-insert library, Sanger sequencing	Lo *et al.* (2007)
Ocean	Archaea and bacteria	6.3 Gbp	Small- and large-insert libraries, Sanger sequencing	Rusch *et al.* (2007)
Mediterranean Sea	Archaea and bacteria	7.184 Mbp	Large-insert library, Sanger sequencing	Martín-Cuadrado *et al.* (2007)
Honey bee	Archaea, bacteria, eukarya, and viruses	NA*	Pyrosequencing	Cox-Foster *et al.* (2007)
Termite hindgut	Bacteria	71 Mbp	Small- and medium-insert library, Sanger sequencing	Warnecke *et al.* (2007)
Human gut	Archaea and bacteria	727 Mbp	Small-insert library, Sanger sequencing	Kurokawa *et al.* (2007)
Coral	Archaea, bacteria, eukarya, and viruses	32 Mbp	Pyrosequencing	Wegley *et al.* (2007)
Soil	Viruses	NA*	Small-insert library, Sanger sequencing	Fierer *et al.* (2007)
Coastal seawater	Bacterioplankton	*c*. 29 Mbp	Pyrosequencing	Mou *et al.* (2008)
Indoor air	Archaea, bacteria, eukarya, and viruses	*c*. 80 Mbp	Small-insert library, Sanger sequencing	Tringe *et al.* (2008)
Ocean	Viruses	NA*	Small-insert libraries, Sanger sequencing	Williamson *et al.* (2008)
Subterranean, hypersaline ponds, marine, freshwater, coral, microbialites, fish, terrestrial animals, mosquito	Archaea, bacteria, eukarya, and viruses	*c*. 1.5 Gbp	Pyrosequencing	Dinsdale *et al.* (2008a)
*Riftia pachyptila*	‘*Candidatus* Endoriftia persephone’ endosymbiont	*c*. 45 Mbp	Small-insert library, Sanger sequencing	Robidart *et al.* (2008)
Coral atolls	Archaea, bacteria, eukarya, and viruses	NA*	Pyrosequencing	Dinsdale *et al.* (2008b)
Activated sludge	‘*Candidatus* Cloacamonas Acidaminovorans’	1.2 Gbp	Large-insert library, Sanger sequencing	Pelletier *et al.* (2008)
North Pacific subtropical gyre	Archaea, bacteria and viruses	45 Mbp (DNA) and 14 Mbp (cDNA)	Pyrosequencing of DNA and cDNA	Frias-Lopez *et al.* (2008)
Yellowstone hot springs	Viruses	30 Mbp	Small-insert library, Sanger sequencing	Schoenfeld *et al.* (2008)
Peru Margin subseafloor sediments	Archaea and bacteria	61.9 Mbp	MDA followed by pyrosequencing	Biddle *et al.* (2008)
Controlled coastal ocean mesocosm	Archaea, bacteria, and viruses	323 Mbp	Pyrosequencing of DNA and MDA-amplified cDNA	Gilbert *et al.* (2008)

* Details not available.

MDA, multiple displacement amplification

Apart from random shotgun sequencing of microbial communities, more targeted approaches involve high-throughput sequencing of individual genomes. Single-cell genomics is based on multiple-displacement whole genome amplification (recently reviewed by Lasken, 2007; Binga *et al.*, 2008; Ishoey *et al.*, 2008). This method has resulted in up to 75% of the expected genome coverage as compared with the standard Sanger sequencing of isolates. The most successful application of single-cell sequencing to date relied on microfluidic cell separation and resulted in the sequencing of at least 1500 genes from a representative of the previously uncharacterized TM7 lineage (Marcy *et al.*, 2007). Other alternatives for dissection of complex samples into simpler components include flow cytometry and cell sorting based on FISH (Raghunathan *et al.*, 2005; Kalyuzhnaya *et al.*, 2006), micromanipulation to isolate a cohesive population, for example *Beggiatoa* filaments (Mussmann *et al.*, 2007), or microbial ‘bait’ to pull out syntrophic assemblages (Pernthaler *et al.*, 2008). Although these approaches provide detailed information about the selected microorganisms, the manipulation removes the environmental setting of the organism and it will omit potentially important co-occurring microorganisms from the analysis.

The wealth of community genomic information allows microbial ecologists to explore the enormous genetic diversity contained within different microbial habitats. However, unless a cell selection method is used, the extent of genomic coverage of community constituents mainly depends on the microbial diversity contained within an analyzed sample. Currently, metagenomic investigations may be broadly classified according to two types: (1) gene-centric investigations where extensive genomic assemblies are unobtainable due to extensive microbial diversity within the sample (e.g. Tringe *et al.*, 2005) and/or due to the sequencing method used (e.g. Edwards *et al.*, 2006) and (2) genome-centric studies where extensive *de novo* assembly is obtainable due to limited species richness (e.g. Tyson *et al.*, 2004), the application of complexity reduction methods (e.g. Pernthaler *et al.*, 2008), or where previously sequenced isolate genomes allow recruitment of genomic fragments (e.g. Coleman *et al.*, 2006).

### Gene-centric metagenomics

Gene-centric approaches using automated gene calling and annotation of genomic fragments followed by the assignment of detected genes to functional categories facilitate the structural and functional comparison of distinct environmental samples. Tringe *et al.* (2005) demonstrated that gene complements vary distinctly between different ecosystems and reflect known characteristics of the terrestrial and marine environments that were sampled, such as photosynthesis in the Sargasso Sea and starch and sucrose catabolism in soil. Environmental gene censuses provide a coarse overview of the genetic potential within a given ecosystem and, by juxtaposition of distinct datasets, can reveal interesting taxonomic and functional aspects of particular habitats.

The gene-centric approach has been applied to a range of different microbial ecosystems. For example, Kurokawa *et al.* (2007) found that the structural and functional composition of infant gut microbiomes varies extensively between individuals and is functionally less redundant compared with adults and children. Overall, the individual gut metagenomes exhibited prominent enrichment in genes indicative of distinct nutrient acquisition strategies related to the hosts' diets. Differences in community composition and functional gene complements are also observed on a large scale, such as in microbial communities inhabiting the water column overlying four coral atolls along an *c*. 750-km-long ocean transect (Dinsdale *et al.*, 2008b). Moving along the transect from a pristine atoll to increasingly human-impacted reefs, Dinsdale *et al.* (2008b) observed a marked shift in community composition and functional gene complement from autotrophy to heterotrophy that may be directly related to anthropogenic effects. DeLong *et al.* (2006) sequenced large-insert libraries derived from microbial communities sampled at different depths in the North Pacific Gyre, and noted the enrichment of particular gene categories in distinct environments, which they hypothesized to reflect distinct adaptive strategies. For example, genes involved in chemotaxis were enriched in the photic zone, suggestive of a free-swimming lifestyle, while deep-water samples were enriched in genes involved in particle attachment and biofilm formation. The broadest overview of differing genetic potentials within microbial communities was recently described across 45 different microbial habitats (Dinsdale *et al.*, 2008a). The study focused on the microbial and corresponding viral constituents of samples from multiple environments ranging from solar salterns to mosquito guts (Dinsdale *et al.*, 2008a). Although most of the functional diversity was redundant, the relative abundances of genes linked to particular metabolisms varied, and as previously highlighted by Tringe *et al.* (2005), the differences in functional gene content reflected the environments from which the samples had been taken.

The relatively new field of experimental metagenomics has so far used a gene-centric approach, but explicitly addresses differences between manipulated systems. Two of the most notable of these types of studies involved comparisons of the gut microbiota of obese and lean mice (Turnbaugh *et al.*, 2006) and the identification of large niche breadth associated with the use of a range of different carbon compounds in the coastal ocean (Mou *et al.*, 2008).

Gene-centric analyses are constrained due to the large fraction of genes of unknown function and the inability to place individual genes into genomic context. The sequencing method used can also significantly bias gene identification, as short reads generated with 454 pyrosequencing are less likely to match distant homologs with blast than reads generated with Sanger sequencing (Wommack *et al.*, 2008). Hence, a subset of fine-scale genetic differences that may be ecologically significant is not considered. In the present review, we focus mainly on genome-centric community genomics because these approaches allow us to infer the effects of fine-scale evolution (recombination, mutation) on community-level ecology and, hence, facilitate a distinctly different view of community composition and function. We refer the reader to the recent review of Raes & Bork (2008) for a more involved discussion of the integration of gene-centric methods with other systems-level data.

### Genome-centric metagenomics

Genome-centric approaches based on extensive genomic reconstruction of community constituents have been applied to microbial ecosystems containing low species richness (e.g. Tyson *et al.*, 2004; Woyke *et al.*, 2006; Robidart *et al.*, 2008) and/or dominant organism types (e.g. García-Martín *et al.*, 2006; Strous *et al.*, 2006). Gene annotation of genomic fragments assigned to specific organisms facilitates comprehensive metabolic reconstructions of community members (García-Martín *et al.*, 2006; Strous *et al.*, 2006; Robidart *et al.*, 2008) and, hence, provides insight into possible metabolic partitioning among community members (Tyson *et al.*, 2004; Woyke *et al.*, 2006; Warnecke *et al.*, 2007). Detailed metabolic reconstructions may reveal new aspects of the metabolisms of certain community constituents and highlight previously unknown characteristics of a particular metabolic process. Tyson *et al.*, (2004) identified nitrogen fixation genes on a genomic scaffold assigned to *Leptospirillum* group III and this organism was obtained in pure culture using nitrogen fixation as an isolation strategy (Tyson *et al.*, 2005). Strous *et al.* (2006) identified candidate genes involved in ladderane biosynthesis and hydrazine metabolism in the composite genome of the dominant organism ‘*Candidatus* Kuenenia stuttgartiensis’, an uncultured *Planctomycete* that carries out anaerobic ammonium oxidation (anammox). These previously unknown genes are important components of the anammox process.

Apart from enabling comprehensive metabolic reconstructions of community members, genome-centric metagenomics allows the fine-scale resolution of genetic heterogeneity within distinct populations. Community genomic studies that achieve extensive *de novo* genomic assemblies of community constituents reveal that the extent of within-population variation differs widely within ecosystems (Table 2). For example, the frequencies of single nucleotide polymorphisms (SNPs) in populations in the AMD system vary from around 0.08% (*Leptospirillum* group II) to 2.2% (*Ferroplasma acidarmanus*; Tyson *et al.*, 2004). The SNP frequency in four endosymbionts of the marine oligochaete *Olavius algarvensis* range from 0.01% (δ4) to 0.1% (γ1) (Woyke *et al.*, 2006; Table 2).

**Table 2:** Single nucleotide polymorphism (SNP) densities

Organism	SNP density (%)	% of SNPs that are replicated*	Average coverage	Environment	Reference
*Candidatus* Accumulibacter phosphatis	0.0006 (US)/0.002 (OZ)	Replicated only	9.2–17.5 × (US)/5.36−7.68 × (OZ)	Sludge bioreactor	Kunin *et al.* (2008)
*Leptospirillum* group II type UBA	0.004	Replicated only	25 ×	Acid mine drainage	Lo *et al.* (2007)
*Kuenenia stuttgartiensis*	0.007	NS†	22 ×	Anammox bioreactor	Strous *et al.* (2006)
*O. algarvensis* symbiont δ4	0.01	Replicated only	3.3 ×	Gutless marine worm	Woyke *et al.* (2006)
*O. algarvensis* symbiont γ3	0.04	Replicated only	5.2 ×	Gutless marine worm	Woyke *et al.* (2006)
*O. algarvensis* symbiont δ1	0.08	Replicated only	8.4 ×	Gutless marine worm	Woyke *et al.* (2006)
*Leptospirillum* group II type 5-way CG‡	0.09	38	20 ×	Acid mine drainage	Simmons *et al.* (2008)
*O. algarvensis* symbiont γ1	0.1	Replicated only	3 ×	Gutless marine worm	Woyke *et al.* (2006)
‘Iplasma’	0.27	12	20 ×	Acid mine drainage	Unpublished data
*Candidatus* Endoriftia persephone	0.29§	NS†	18.6 ×	*Riftia pachyptila* symbiont	Robidart *et al.* (2008)
‘Eplasma’¶	0.53	42	10 ×	Acid mine drainage	Unpublished data
*Ferroplasma* type II	2.2	NA∥	10 ×	Acid mine drainage	Tyson *et al.* (2004)
*Ferroplasma* type I	3	NA∥	4.5 ×	Acid mine drainage	Allen *et al.* (2007)
Archaeal virus contig from metagenomic library	7.05	NS†	11 ×	Yellowstone hot springs	Schoenfeld *et al.* (2008)
Archaeal virus AMDV2	27	54	17.5 ×	Acid mine drainage	Andersson & Banfield (2008); unpublished data

* Noted in entry whether all polymorphisms or replicated polymorphisms only were counted.

† Not specified whether all polymorphisms or just replicated polymorphisms were counted.

‡ All bases with phrap sequence quality scores <25 were ignored in the polymorphism calculation.

§ Calculated only for a subset of genes.

¶ Partial assembly.

∥ Details not available.

### Genetic heterogeneity within microbial populations

The genetic heterogeneity of microbial populations was first apparent from the comparison of multiple genome sequences from organisms considered to be strains of the same species. At first, the observation of 25% unique gene content between *Escherichia coli* K12 and O157:H7 despite *c*. 98% average nucleotide identity (ANI) between their orthologs seemed remarkable (Hayashi *et al.*, 2001). These findings were confirmed by the comparison of 20 available strains of *E. coli* and *Shigella* sharing 98–99% ANI, which predicted that every newly sequenced genome will add *c*. 300 new genes to the *E. coli*‘pan-genome’ (Konstantinidis *et al.*, 2006). The pan-genome size seems to depend on the ecology of the organism. Phenotypically and ecologically more coherent species, such as obligate pathogens, tend to have smaller pan-genomes [<50 genes added for every new strain of *Streptococcus agalactiae* (Tettelin *et al.*, 2005)] than organisms residing in more dynamic environments (Rocap *et al.*, 2003; Thompson *et al.*, 2005). Population-level heterogeneity even exists within supposedly clonal populations used for sequencing, mostly due to rapid processes including the spread of insertion sequence elements and phase inversions (Cerdeno-Tarraga *et al.*, 2005; Chain *et al.*, 2006). Overall, the findings indicate the dynamic nature of population-level genome content and structure.

The extent of heterogeneity within bacterial and archaeal populations calls into question whether our current species definition corresponds with distinct evolutionary units or natural groups (Doolittle & Papke, 2006; Bapteste & Boucher, 2008). Higher-level taxonomic groups based on phylogenetic markers are demonstrably coherent despite extensive strain-to-strain variation (Ochman *et al.*, 2005), possibly because differences in gene content are localized on genomic islands (Chain *et al.*, 2006; Coleman *et al.*, 2006; Kettler *et al.*, 2007; Mathee *et al.*, 2008). These islands, which might be neutral or transient, mainly encode hypothetical proteins (Konstantinidis *et al.*, 2006). Nevertheless, a subset may confer adaptive traits. We will revisit the question of the fitness effects of gene content variation later in this review.

Because reliance on isolate genomes alone limits the scope of observable genomic heterogeneity, recent efforts have focused on using random shotgun sequencing of microbial communities followed by assembly and documentation of various types of within-population variability. Genome-centric approaches fall into two classes: (1) *de novo* sequence assembly and (2) recruitment of environmental genome fragments to isolate genomes followed by some degree of assembly.

#### *De novo* assembly

**Fig. 2:**
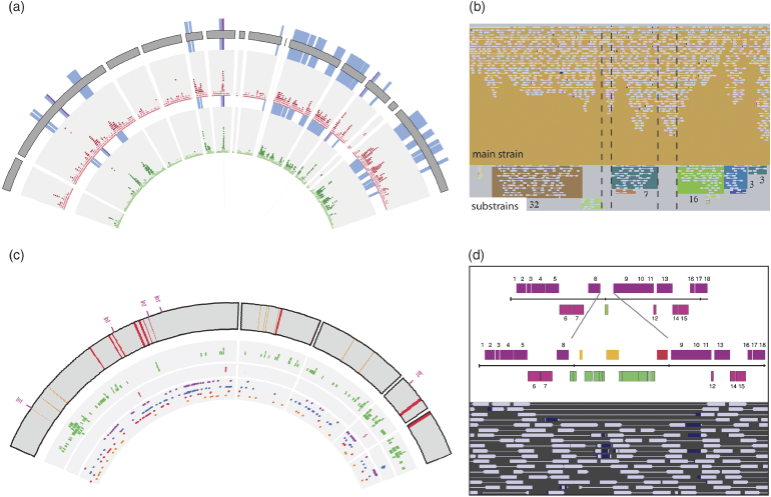
Examples of genome-wide fine-scale analysis of sequence variation in *Leptospirillum* group II 5-way CG. (a) Part of the *Leptospirillum* group II 5-way CG genome assembled from population genomic data. The first inner ring shows a moving average of SNP density. Dark red indicates local SNP density of >0.5%, while pink indicates <0.5%. The second inner ring shows a moving average of polymorphism frequency (scale 0–0.7%). Light-blue highlights indicate the location of substrains within the 5-way CG population (>99% sequence similarity). Purple highlights indicate the location of deeply sampled reads of more divergent strains incorporated into the population (*c*. 94% sequence similarity). (b) Closeup of the data used to generate the figure in (a). A screenshot of a contig from the program strainer is shown, with individual reads shown as light-gray blocks. Strains defined by shared polymorphisms are shown in distinct colors, with the main strain in orange. The vertical dashed lines indicate regions within the main strain not overlapped by any substrain. (c) Overview of different sources of genomic variation over a 500-kb segment. In the outer ring, tRNAs are indicated with orange, transposons with red, and integrases with ‘Int.’ The location and length of strain variant paths (see main text) are shown in green in the first inner ring, and the locations of recombinant reads are shown in the second inner ring. The innermost ring shows nonsynonymous SNPs in blue, synonymous SNPs in purple, intergenic SNPs in red, and SNPs resulting in frameshifts in orange. The image was generated with circos (M. Krzywinski, http://mkweb.bcgsc.ca/circos/). (d) Gene content variation from an assembly point of view. Alternate genome paths are shown in the top. The uppermost path shows the main genome path, and the bottom path shows the insertion of several genes (colored green, orange, and red). The lower part shows individual sequencing reads, with inserted regions indicated by dark blue. Mate-paired reads on the top line are separated by the presence of the insert.

*De novo* genome assembly from shotgun sequencing data was used to obtain comprehensive and deeply sampled genomic datasets (up to 25 × coverage) for multiple organisms from AMD biofilms (Tyson *et al.*, 2004; Allen *et al.*, 2007; Lo *et al.*, 2007; Simmons *et al.*, 2008), which allowed for a direct analysis of *in situ* population heterogeneity. The level of within-population variability ranges from near-clonal (Lo *et al.*, 2007) to freely recombining (Eppley *et al.*, 2007b; Table 2). The two deepest coverage assemblies were obtained for two *Leptospirillum* group II populations sampled at the UBA and 5-way locations within the Richmond Mine (Lo *et al.*, 2007; Simmons *et al.*, 2008;Fig. 2). These two populations are *c*. 95% identical at the amino acid level, although they have been shown to recombine (Lo *et al.*, 2007; Denef *et al.*, 2008). In addition, there is recombination within the *Leptospirillum* group II 5-way CG population between distinct substrains <0.5% divergent (Simmons *et al.*, 2008; Fig. 2). Strikingly, based on the extensive gene content variation within the *Leptospirillum* group II population, it could be inferred that the number of unique genotypes was only one order of magnitude less than the number of cells in the population (Simmons *et al.*, 2008).

High levels of recombination were detected in two distinct populations (types I and II) related to *F. acidarmanus*. The *Ferroplasma* type II population within one sample consisted of individuals with mosaic genomes formed by recombination between distinct genome types (Tyson *et al.*, 2004). A comparison of a *Ferroplasma* type I isolate with its corresponding populations revealed that much of the observed heterogeneity was due to transposase movement and phage insertions and deletions (Allen *et al.*, 2007). The majority of *Ferroplasma* type I genes were under strong stabilizing selection as only six loci out of 1963 exhibited nonsynonymous vs. synonymous SNP ratios indicative of positive selection. Recombination was more frequent within both *Ferroplasma* type I and type II populations than between them, consistent with a log-linear decline in recombination frequency with sequence divergence (Eppley *et al.*, 2007b). In summary, the AMD system studies have confirmed the isolate sequence-based hypothesis of population-level heterogeneity in gene content and the movement of mobile elements within natural populations. Additionally, these studies uncovered the prevalence of recombination within and between natural populations.

Community genomic approaches have also resulted in deep sequence coverage of the dominant population in two types of activated sludge enrichment cultures (García-Martín *et al.*, 2006; Strous *et al.*, 2006). Little fine-scale variation was apparent in the dominant population of the anammox bacterium ‘*Candidatus* Kuenenia stuttgartiensis’ (Strous *et al.*, 2006). The sludge community was dominated by a single clonal type and this may be due to the long-term selection implemented by enrichment culturing. In contrast, a study using sludge from enhanced biological phosphorus removal (EBPR) reactors in the United States and Australia did retrieve population heterogeneity. These communities were dominated by similar genotypes (>95% identical at the nucleotide level) of ‘*Candidatus* Accumulibacter phosphatis’ (*Accumulibacter phosphatis*; García-Martín *et al.*, 2006), but substantial strain diversity (up to 15% divergent at the nucleotide level) was present within both *A. phosphatis* populations. This heterogeneity was corroborated by extensive fine-scale variation among *A. phosphatis* rRNA internally transcribed spacer regions (He *et al.*, 2006) and the polyphosphate kinase 1 gene (He *et al.*, 2007; Wilmes *et al.*, 2008a).

#### Genomic fragment recruitment

For metagenomic datasets generated from diverse environments where *de novo* assembly is difficult or impossible, the *in silico* recruitment of closely related genomic fragments and comparison with sequenced isolate genomes is an effective approach to study within-population variation. The Global Ocean Survey (GOS) sequencing data, which comprised 6.3 Gbp generated from diverse marine microbial communities along an 8000 km ocean transect, required the extensive use of this method (Rusch *et al.*, 2007). Fragment recruitment, as first described by Coleman *et al.* (2006), was performed for those genera for which isolate sequences were available [*Pelagibacter* (Giovannoni *et al.*, 2005), *Prochlorococcus* (Rocap *et al.*, 2003), and *Synechococcus* (Palenik *et al.*, 2006)]. In addition, newly assembled composite genomic fragments from the GOS data provided additional reference sequence. These analyses revealed tremendous sequence variation consisting of SNPs, gene and genomic island insertions, deletions and rearrangements, and geographic clines in sequence patterns. These results are consistent with the extensive allelic diversity and genome size variation previously observed in marine microbial populations (Thompson *et al.*, 2005).

Mate-pair analysis of the GOS dataset suggested that gene synteny was highly conserved. A more quantitative analysis involving gene-based matching of metagenomic fragments to a *Pelagibacter* isolate genome found that gene synteny was highly conserved between populations, despite large geographic separation and an average 30% amino acid divergence (Wilhelm *et al.*, 2007). It was suggested that gene order conservation is due to low functional diversity in the SAR11 population, with the caveat that large-scale genome rearrangements are less likely to be identified by the applied method. However, a SAR11 fosmid clone from the English Channel exhibited multiple differences in a hypervariable region compared with the previously available SAR11 sequences (Gilbert *et al.*, 2008). Rusch *et al.* (2007) also argue that the fine-scale genetic variation among closely related organisms may reflect functional differentiation between subtypes.

The GOS (Rusch et al., 2007) and Sargasso Sea (Venter et al., 2004) datasets have also been used in additional recruitment studies using reference sequences from other sources, such as the picoeukaryote *Ostreococcus tauri* (Piganeau & Moreau, 2007) and *Cenarcheaum symbiosum* (Hallam *et al.*, 2006). Using the genome of *O. tauri*, Piganeau & Moreau (2007) recruited genomic fragments amounting to 23% of the complete nuclear genome (14% of protein-coding genes), identified two new *Ostreococcus* strains from the recruited fragments and found that introns have a high proportion of conserved sites (70%). The *C. symbiosum* reference sequence was assembled from a limited number of fosmid clones from a sponge sample highly enriched for the target organism (Hallam *et al.*, 2006). Fosmids binned into two subpopulations and were *c*. 15% divergent at the nucleotide level between populations and *c*. 2% divergent within each population. Again, gene order seemed to be conserved between the two subpopulations, as well as between the sponge symbionts and free-living relatives in the Sargasso Sea (based on fragment recruitment). The authors suggested that clonal diversification was the dominant evolutionary process in *C. symbiosum*. Population-level heterogeneity was clearly present, although the lack of sequencing depth weakens conclusions about gene content homogeneity within the symbiont populations. Genomic regions that were not present in the planktonic population were suggested to be essential for the symbiotic interactions of *C. symbiosum* and its sponge host.

A tandem isolate and metagenomic sequencing approach was used by Bhaya *et al.* (2007) on microbial mat communities of Yellowstone hot springs. Two cyanobacterial isolates (*Synechococcus* OS-A and OS-B') that dominate the microbial mats at different temperatures were sequenced. Both *Synechococcus* population representatives shared a large proportion of their gene content at high identity but exhibited extensive genome rearrangements. Differences in phosphate and nitrogen pathways indicated that both populations are distinct in their nutrient utilization. The two isolate genomes served as ‘anchor’ genomes to recruit closely related metagenomic sequences. These exhibited a high degree of variability and demonstrated that the sequenced isolates are not representative of all *Synechococcus* populations at the two sites. Interestingly, the low-temperature populations exhibited greater sequence diversity compared with the high-temperature populations. Furthermore, Bhaya and colleagues found evidence for functionally specialized populations and, hence, suggest that these ‘ecotypes’ occupy distinct niches within the microbial mats.

A recruitment-based comparative metagenomic approach was also applied to the halophilic square archaeon *Haloquadratum walsbyi*. This organism, which dominates mature saturated brine communities, has only recently been isolated and sequenced (Bolhuis *et al.*, 2006). End-sequence analysis of a metagenomic fosmid library revealed a remarkable diversity of genes and evidence for genomic islands (Legault *et al.*, 2006; Cuadros-Orellana *et al.*, 2007), leading to the suggestion that the pan-genome of *H. walsbyi* may be at least double the size of the sequenced isolate. Some genomic islands displayed features of virus-mediated genetic exchange. Importantly, the vast majority of dissimilar gene content was related to small-molecule transport and detection, representing possible adaptations to different pools of organic nutrients (Cuadros-Orellana *et al.*, 2007).

In summary, most observational studies, either based on comparative genomic analysis of isolates or metagenomic datasets, consistently reveal within-population gene content and sequence diversification. These findings substantiate previous work using phylogenetic marker genes and genome fingerprinting of *Vibrio* isolates that showed extremely high diversity between closely related strains (Acinas *et al.*, 2004; Thompson *et al.*, 2005). The emerging picture is of populations as clouds of genetic material separated from other related populations by levels of genetic exchange that decline with increasing sequence divergence (Eppley *et al.*, 2007b; Rusch *et al.*, 2007; Simmons *et al.*, 2008; G. J. Dick *et al.*, unpublished data). The level of genetic exchange and sequence divergence varies from little (near-clonal) to high (free recombination), as measured both by population genomics and more traditional multilocus sequence typing (MLST) of isolates (reviewed by Pérez-Losada *et al.*, 2006). The set of variable genes and genome rearrangements may be so large in some populations that no two individuals have exactly the same genotype (Thompson *et al.*, 2005; Rusch *et al.*, 2007; Chantratita *et al.*, 2008; Simmons *et al.*, 2008). Interpretation of extensive genetic heterogeneity is generally unresolved. Possible explanations for high levels of variation include diversification on the generation timescale in response to viral predation (Andersson & Banfield, 2008; Tyson & Banfield, 2008), neutral diversification (Acinas *et al.*, 2004) or resource partitioning between closely related strains [<1% 16S rRNA gene divergence (Hunt *et al.*, 2008)]. We discuss below some methods that can be applied to the question of whether within-population variation is largely neutral or has adaptive significance.

## Evolutionary interpretation of population heterogeneity

Interpreting the adaptive significance of sequence variation within and between populations represents a considerable challenge, which is only beginning to be addressed with the advent of community genomic data. Much of the observed variation may be neutral, and persist in microbial populations due to potentially quite large, but presently unknown, effective population sizes (Mes, 2008). Basic population genetic theory predicts that neutral variation will persist in a population for a number of generations of the same order of magnitude as the effective population size, *N*_e_, if genetic drift is the only force acting on it (Gillespie, 2004; Mes, 2008). *N*_e_ determines the rate at which variation is lost from a population, and is highly sensitive to bottlenecks (such as periodic selection events). Given the enormous census sizes of microbial populations, however, *N*_e_ could still be large enough to ensure an extremely long fixation time for neutral variation. In fact, the *N*_e_ for *E. coli* is estimated to be 10^8^–10^9^ based on polymorphism at the third codon position (Hartl *et al.*, 1994). One theoretical model suggests that mutation occurring in neutral gene variants is sufficient to block their fixation in large populations, leading to a large flux of transient novel sequences (Berg & Kurland, 2002). This is consistent with empirical observations of high genotype diversity derived from comparisons of isolates (Thompson *et al.*, 2005) and population genomic assemblies (Allen *et al.*, 2007; Simmons *et al.*, 2008).

**Fig. 3:**
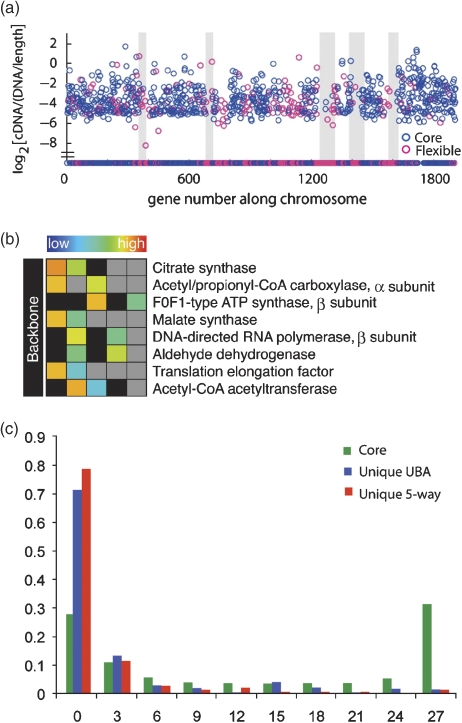
Experimental evidence of the role of the ‘flexible’ genome content. (a) Environmental transcriptomic data from *Prochlorococcus* MIT3901 from a Sargasso Sea sample (Frias-Lopez *et al.*, 2008). The cDNA levels, normalized using the levels of DNA found in the same sample, are shown for all identified genes of this particular strain (‘core genome’ genes present in all *Prochlorococcus* genomes: blue; ‘flexible genome’ genes present in at least one but not all genomes: pink). Hypervariable regions are highlighted with gray bars. While many ‘flexible’ genes are expressed, genes located in the hypervariable regions are underrepresented. (© 2008 The National Academy of Sciences of the USA). (b) Heterogeneous protein expression within activated sludge dominated by ‘*Candidatus* Accumulibacter phosphatis’ (*Accumulibacter phosphatis*; Wilmes *et al.*, 2008a, b). Orthologous proteins (90% amino acid identity; represented by individual blocks) from the US Phrap assembly (García-Martín *et al.*, 2006) aligned against the *A. phosphatis* composite genome that serves as the backbone. Unique spectral counts (identified peptides specific to a certain protein variant) heat-mapped onto the alignment (gray blocks indicate absence of orthologs; black blocks indicate no unique peptide spectra identified). (c) Summary of the expression data of the *Leptospirillum* group II population from 27 samples from the Richmond Mine (Iron Mountain, CA) as determined by proteomics (V.J. Denef *et al.*, unpublished data). The fraction of proteins never identified (0), identified in 1–3 samples (3), 4–6 samples (6), etc. are shown. Clearly, the unique genes [as determined from comparative genomic analysis of the two available genomes, UBA-type (blue) and 5-way CG type (red)] are expressed in significantly fewer samples than the core genome complement (green).

Expression and bioinformatic studies have provided indirect insight into the differential fitness of genotypic variants. Hypervariable regions, often called ‘gene islands,’ contain a significantly higher proportion of novel genes compared with the rest of the genome (Hsiao *et al.*, 2005). While in general, a lower fraction of genes in islands are expressed as compared with genes in the core genome, some can be among the most abundant transcripts or proteins in environmental samples (Ram *et al.*, 2005; Frias-Lopez *et al.*, 2008; V.J. Denef *et al.*, unpublished data; D.S.A. Goltsman *et al.*, unpublished data; Fig. 3). The size of the expressed fraction seems to vary depending on the organism studied, with the caveat that there are very few studies of this type available.

Analysis of environmental transcripts extracted from a marine sample showed that a majority of the flexible gene content of *Prochlorococcus* genomes was both present and expressed at similar levels to core genes (Frias-Lopez *et al.*, 2008; Fig. 3a). Laboratory experiments with isolated strains of *Prochlorococcus* also support the importance of hypervariable regions in environmental adaptation. In one strain, 26% of all genes in highly variable regions of the genome were differentially expressed under changed nutrient or light conditions in culture (Coleman *et al.*, 2006). Bioinformatic analysis also supports the potential adaptive value of genomic islands in other species. For example, several of the genomic islands differentiating the soil bacterium *Burkholderia xenovorans* LB400 from other strains of its species contain the genes enabling it to degrade chlorinated aromatics (Chain *et al.*, 2006). Many additional examples regarding the importance of genomic islands in environmental adaptation have been summarized elsewhere (Dobrindt *et al.*, 2004).

Proteomics holds particular promise for the elucidation of discrete functional differences between closely related organisms and placing these into evolutionary and environmental context. Distinct protein profiles for strains of the same species are easily obtained by single-dimensional (Vauterin *et al.*, 1991) and two-dimensional (Dopson *et al.*, 2004) polyacrylamide gel electrophoresis. Using protein profiles from four *Ferroplasma* isolated strains that are >98.9% similar at the 16S rRNA gene level but exhibit phenotypic differences in culture, Dopson *et al.* (2004) constructed a phylogenetic tree that was congruent with a tree based on DNA–DNA similarities and, thus, demonstrated the ability of using proteomics for phylogenetic characterization of discrete populations. Morris *et al.* (2007) were able to deduce the contribution of distinct strains of *Dehalococcoides* spp. to anaerobic dehalogenation within an uncharacterized mixed culture by determining the relative abundances of strain-specific peptides obtained from reductive dehalogenases. With the advent of shotgun proteomics based on liquid chromatography coupled with high-resolution tandem mass spectrometry and its application to microbial communities, individual peptides that originate from discrete populations within a mixed microbial community are identified (Lo *et al.*, 2007; Wilmes *et al.*, 2008a). By assigning peptides to different populations, Lo *et al.* (2007) were able to infer the genome architecture of a single *Leptospirillum* group II population within a genomically uncharacterized sample and demonstrated that its genome is a hybrid formed by recombination of the UBA and 5-way CG genome types. In the AMD biofilm system, *Leptospirillum* group II genome types are tractable because distinct biofilm samples are limited in their genotypic diversity (Denef *et al.*, 2008). Although the picture becomes complex when several strains of the same species co-occur, strain-specific contributions to the overall protein pool can still be resolved.

Strain-resolved proteomics has been used to differentiate the expression of co-occurring protein variants within a single sample of activated sludge cultivated for EBPR in the United Kingdom and dominated by *A. phosphatis* (Wilmes *et al.*, 2008a; Fig. 3b). The study revealed that 59% of identified proteins were derived from the flanking *A. phosphatis* populations and not from the dominant *A. phosphatis* strain in the sequenced sludges. A significant subset of these was involved in core-metabolism and EBPR-specific pathways. These results suggest an essential role for genetic diversity in maintaining the stable performance of microbial community-based biotechnological systems.

Somewhat different dynamics are apparent in AMD biofilm communities, where both proteomic (V.J. Denef *et al.*, unpublished data) and genomic studies (Allen *et al.*, 2007; Simmons *et al.*, 2008) so far do not support large fitness effects for regions of variable gene content. The two *Leptospirillum* group II sequence types dominating the Richmond Mine AMD system differ by only 0.3% at the 16S rRNA gene level, and 20% of each organism's genome is unique relative to the other (Lo *et al.*, 2007). An extensive analysis of 27 environmental proteomes derived from biofilm samples taken from a variety of environmental conditions has shown that while *c*. 70% of the proteins encoded by genes shared between organisms were identified, *c*. 75% of the unique gene complement was never identified, and only 1% of unique proteins were identified in every sample (V.J. Denef *et al.*, unpublished data; Fig. 3c). In summary, if we take expression levels under different conditions as an indicator of fitness, some proportion of genes in variable regions may have adaptive value, but others appear to be largely neutral. Possible caveats to this include the possibility that proteins expressed at low levels could significantly affect fitness, and methodological limitations of expression measurements, such as poor sensitivity or biases such as the low identification rate of membrane proteins. Nonetheless, the significantly lower identification levels for unique genes do strongly suggest that most of them are transient and do not significantly affect organismal fitness. Additional studies are clearly required to further address this issue.

Detection limits for community proteomics suggest that each organism for which a protein is identified must be present at an abundance of at least a few percent of the total community (N.C. VerBerkmoes *et al.*, unpublished data), and the range of detectable proteins will improve with future technical developments in proteomics (P. Wilmes *et al.*, unpublished data). To evaluate whether the expressed variants are important for community function, it will be necessary to measure expression levels over time in conjunction with process measurements. In addition, structural studies of microbial communities (e.g. biofilms; Wilmes *et al.*, 2008b) may show whether particular variants are localized within distinct microniches. For example, enzyme variants that may be the most suited for a particular biotechnological application may be located at a particular position along a chemical gradient. Hence, more fine-scale measurements will be necessary in future to resolve the functional significance of genetic heterogeneity within microbial communities.

### Sequence clusters in population genomics

Defined sequence clusters have been identified in metagenomic assemblies by binning, assembly based on sequence homology, or identity to large fragments of known origin, as discussed above (Tyson *et al.*, 2004; Hallam *et al.*, 2006; Allen *et al.*, 2007; Eppley *et al.*, 2007b; Rusch *et al.*, 2007; Simmons *et al.*, 2008). Smaller, less-divergent sequence clusters within assemblies can be detected through manual analysis of shared, linked polymorphisms (Whitaker & Banfield, 2006; Eppley *et al.*, 2007b). Recent work shows that tetranucleotide frequencies can be used to cluster reads and contigs derived from complex natural communities at the species to genus level and higher, but they do not differentiate between closely related species, despite likely ecologically distinct roles (G. J. Dick *et al.*, unpublished data).

The existence of these clusters, which are also apparent in isolate-based MLST studies, indicates that genetic exchange between populations is limited to varying degrees. It is unclear, however, as to how sequence clusters correspond to microbial ‘species’ (Achtman & Wagner, 2008) or ecologically distinct populations (Whitaker & Banfield, 2006). Possible processes leading to clusters include adaptation to particular environmental niches among coexisting populations, physical isolation or a decline in recombination frequency between coexisting populations due to neutral divergence within genomes (Whitaker *et al.*, 2005; Fraser *et al.*, 2007) without invoking fitness differences (Fraser *et al.*, 2005, 2007; Falush *et al.*, 2006). In one model of speciation (Fraser *et al.*, 2007), the degree of clustering depends on the level of recombination relative to mutation. When recombination is low, populations have a largely clonal structure; sequence clusters continually emerge, split, and disappear over time. Distinct clusters disappear when recombination rates are one quarter to twice the mutation rate, marking the transition from a clonal to sexual population structure (Fraser *et al.*, 2007). Because the rate of homologous recombination in bacteria is known to decline with increasing sequence divergence (Majewski, 2001), genetic drift could potentially lead to reduced rates of within-cluster relative to between-cluster recombination sufficient to cause the emergence of new species. The plausibility of this process appears to depend strongly on the dependence of the recombination rate on sequence divergence, population size, and other modeling assumptions, but under some reasonable parameter schemes, it is at least possible (Falush *et al.*, 2006; Fraser *et al.*, 2007).

**Fig. 4:**
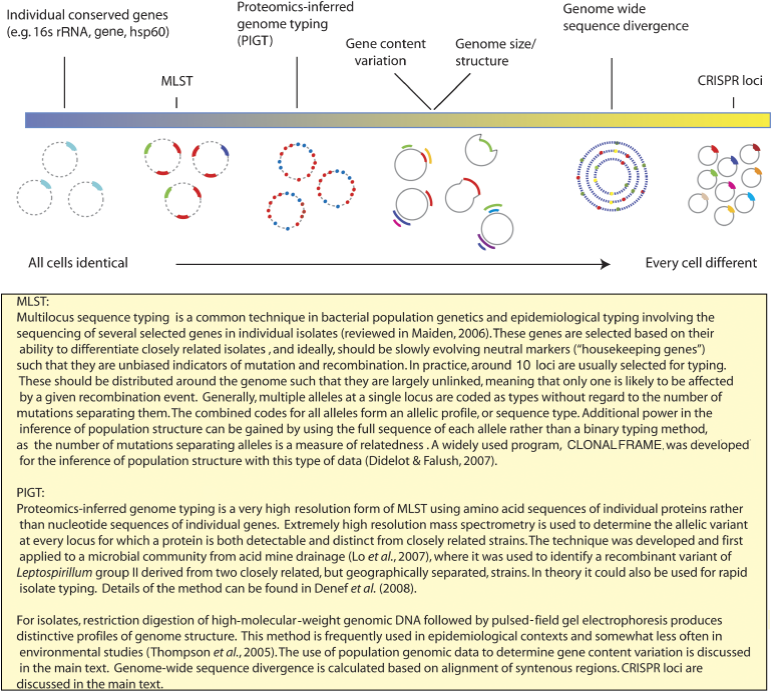
Continuum of variation with box text.

Much of the theoretical literature on the formation of sequence clusters (e.g. Spratt *et al.*, 2001; Fraser *et al.*, 2005; Hanage *et al.*, 2006; Didelot & Falush, 2007) is based on MLST data (Fig. 4), which are used to estimate rates of recombination, mutation, and migration. It is worth keeping in mind, however, that MLST allelic profiles subsume levels of variation detectable with higher resolution methods (Fig. 4), for example, strains of *Vibrio splendidus* differing at <1% of their 16S rRNA gene sequences showed large genome size differences (Thompson *et al.*, 2005), and strains of *Burkholderia pseudomallei* found to be identical by MLST showed variable pulsed-field gel electrophoresis banding patterns (Chantratita *et al.*, 2008). The clustered regularly interspaced short palindromic repeats (CRISPR) locus involved in phage resistance shows the most extreme level of fine-scale heterogeneity reported to date. In fact, it has been suggested that each cell within the *Leptospirillum* group II population has a distinct CRISPR locus (Tyson & Banfield, 2008). These levels of genome-wide variation have not been fully incorporated into evolutionary models for cluster formation and speciation.

### Evolutionary models

It is useful to take a brief look at how the extensive population genetic and experimental literature on microbial evolution might inform our understanding of population genomic data. The classical model of microbial evolution is the ‘periodic selection model,’ which was supported by early experimental work in *E. coli* (Atwood *et al.*, 1951) and has a long history in bacterial population genetics (e.g. Levin, 1981). Briefly, this model posits that beneficial mutations with large effects on fitness arise rarely in asexual populations. The individual containing this large effect mutation rapidly rises to fixation via a selective sweep. Because recombination is essentially absent, this sweep carries an entire genotype to fixation, erasing diversity at all other loci. During the period of stasis in between the appearance of large-effect mutations, neutral diversity can again accumulate at multiple loci.

The periodic selection model is the basis for the ‘clonal ecotype’ model proposed by Cohan and others (Cohan, 2006; Ward, 2006). According to this model, in an environmental context, a single clonal type occupies a particular niche. This comes about because mutations that lead to increased fitness in the niche periodically arise in the population, leading to selective sweeps and the loss of neutral diversity. Multiple sequence clusters are inferred to represent occupants of distinct niches or, alternatively, the mixing of two physically separated populations. This model is rarely tested directly (but see Simmons *et al.*, 2008). Typically, one or more marker gene phylogenies are constructed and the clustering of particular phylogenetic groups according to a limited set of environmental parameters is tested. A positive correlation is interpreted as a support for the ecotype model (Ward, 2006; Koeppel *et al.*, 2008; Ward *et al.*, 2008) because it implies that sequence clusters correspond to ecologically distinct populations.

The periodic selection model assumes that beneficial mutations are rare enough that they will not occur simultaneously in multiple individuals within a population, which may not be correct. The clonal interference model describes the dynamics of evolution when different beneficial mutations occur in multiple individuals before one of them can rise to fixation. Competition between these individuals results in the loss of some mutations and delayed fixation of others (Gerrish & Lenski, 1998). Clonal interference has been shown to occur in laboratory populations of *E. coli*, resulting in less-effective periodic selection (de Visser & Rozen, 2006). The amount of standing variation within a sequence cluster is probably larger under a clonal interference regime than a simple periodic selection regime, but because only one of these multiple mutations ultimately fixes in the population, marker gene phylogenies are insufficient to distinguish the two alternatives. Recent theoretical and experimental work suggests that multiple beneficial mutations co-occur in a subset of individuals within a population, and that these high fitness individuals drive the overall rate of evolution (Desai *et al.*, 2007). Because smaller effect mutations are not lost in this regime, the amount of standing variation in the population will probably be higher than under either the clonal interference or the periodic selection models, but the form it would take in population genomic data is not known.

Theoretical and experimental work suggests that recombination provides a fitness advantage in microbial populations, which scales with the mutation rate (Cooper, 2007), suggesting that the clonal models described above may not be appropriate in all circumstances. In fact, high intraspecific recombination rates are frequently observed in environmental microbial populations using both MLST of isolates (Vergin *et al.*, 1998; Papke *et al.*, 2004; Whitaker *et al.*, 2005; Vos & Didelot, 2008) and population genomic (Tyson *et al.* 2004; Allen *et al.*, 2007; Eppley *et al.*, 2007b; Simmons *et al.*, 2008) and proteomic (Lo *et al.*, 2007; Denef *et al.*, 2008) data. Recombination unlinks the evolutionary fate of different parts of a genome, allowing selection to operate independently on individual loci or sets of linked loci. If selection is relatively weak, the net effect is higher levels of standing diversity within a population than we would expect from the clonal models discussed above. If recombination in a population is extensive, phylogenetic signals of vertical descent can be obscured. In fact, incongruence between phylogenies derived from different loci within a population is a widely used indicator for the occurrence of recombination (Feil & Spratt, 2001). Recombination plus weak selection can therefore result in the appearance of sequence clusters that do not correspond to ecologically unique species (Cohan, 2006; Whitaker & Banfield, 2006). We discuss below some methods that can be used to detect recombination directly in population genomic data.

## Application of population genetic techniques to metagenomic data

The challenges inherent to the analysis of metagenomic data have not yet sparked the widespread development of novel theoretical methodology in the population genetic community (but see Johnson & Slatkin, 2006, 2008). Additionally, most population genomic studies do not make use of existing methods, apart from the basic calculation of polymorphism frequency in assemblies (Table 2). In general, existing population genetic tests are derived from theoretical models that predict how variation is distributed within and between individuals in a population and are based on assumptions about the evolutionary process. Through the analysis of sequence variation, these models attempt to calculate rates of mutation, selection, and recombination. Population genomic data from microbial communities present a unique challenge to such methods, in that each individual sequencing read is most likely derived from an individual cell. Genomic contigs produced through automated or manual assembly are composite sequences derived from multiple individuals and cannot be assumed to correspond to any real sequence in a population (Fig. 2). Especially in short-insert sequencing libraries, this means we cannot physically reconstruct the genome of any individual cell (a haplotype). Statistical reconstructions of individual haplotypes may be possible based on correlations in polymorphism frequency between samples, but such methods do not yet exist.

The lack of haplotype information presents particular problems for methods designed to detect recombination through comparisons of sequences from different individuals, using the coalescent theory (e.g. McVean *et al.*, 2002; Fearnhead *et al.*, 2004) or phylogenetic break-point methods (Minin *et al.*, 2005). The assumptions of these methods allow recombination detection only on length scales smaller than an individual clone. This limitation makes any model-based detection of recombination over longer length scales or in less-variable genomes difficult. Hence, the only studies to tackle the problem of measuring recombination rates in large-scale population genomic datasets have done so using manual identification of breakpoints, which require a polymorphism density high enough for visual detection (Whitaker & Banfield, 2006; Eppley *et al.*, 2007b; Simmons *et al.*, 2008). This approach revealed a log-linear decline in recombination frequency with sequence divergence between populations of the archaeaon *Ferroplasma* present in AMD, consistent with findings in isolate genomes (Eppley *et al.*, 2007b). Putative recombination breakpoints between very closely related strains of the bacterium *Leptospirillum* group II type 5-way CG (>99.5% relatedness) were identified with the visualization program strainer (Eppley *et al.*, 2007a), but due to the low overall polymorphism density, their exact location could not be defined (Simmons *et al.*, 2008). Recombination breakpoints were also identified in *Leptospirillum* group II using strain-resolved shotgun proteomics (Lo *et al.*, 2007). It should be noted that recombination is also identifiable in population genomic datasets through discordant phylogenies for individual genes (Whitaker & Banfield, 2006).

The analysis of selection in individual genes, indels, or intragenic regions pulled out from population genomic datasets is more straightforward, and has been applied in a number of population genomic studies (e.g. Zeidner *et al.*, 2005; Allen *et al.*, 2007; Piganeau & Moreau, 2007; Wilhelm *et al.*, 2007). Nielsen (2005) provides an excellent nontechnical overview of methods to detect selection in sequence data. Briefly, for individual genes, these fall into two classes: frequency spectrum and neutral/nonneutral mutations. The first tests whether the frequency distribution of polymorphisms in a set of aligned sequences is consistent with positive, negative, or no selection under particular evolutionary models. The second involves a comparison of the number of synonymous substitutions (assumed to be neutral) with the number of nonsynonymous substitutions (assumed to have a fitness effect). A d*N*/d*S* ratio >1 for the whole gene is generally assumed to indicate positive selection, because nonsynonymous substitutions would not be retained in the population unless they increased individual fitness. Caveats to this method include a systematic bias in comparisons of closely related organisms (Rocha *et al.*, 2006) and a lack of power to detect selection when it occurs only on a subset of sites within a gene. In fact, most large-scale studies of d*N*/d*S* detect negative selection (the reduction of genetic diversity due to the elimination of deleterious mutations) on nearly all genes (Allen *et al.*, 2007; Petersen *et al.*, 2007). More complex phylogenetically based methods need to be used to detect particular sites under selection within a gene (Yang & Swanson, 2002). This is important to note, as single nonsynonymous mutations can alter the kinetics and specificity of enzymes, providing a means for the adaptation of distinct strains to specific environmental conditions. For example, a single amino acid substitution can switch marine proteorhodopsins (a widely distributed light-driven proton pump) from blue light to green light absorbing (Kelemen *et al.*, 2003; Man *et al.*, 2003), and this point mutation allows spectral tuning according to the position along a depth-dependent light gradient (Béjà*et al.*, 2001).

The McDonald–Kreitman (MK) test (McDonald & Kreitman, 1991) is a more powerful use of counts of synonymous and nonsynonymous data. This test posits that under a model of neutral evolution, the ratio of nonsynonymous to synonymous substitutions within a population is the same as the ratio of nonsynonymous to synonymous fixed differences between populations. An excess of replacement fixed differences indicates positive selection on a particular locus, whereas a dearth indicates negative selection. This test is particularly well suited for testing the ‘ecotype’ hypothesis (Ward *et al.*, 2008). This hypothesis predicts that regions differentiating coexisting organisms should encode genes responsible for their increased fitness in particular niches. If these regions are orthologous, and each coexisting organism is uniquely adapted to a particular niche, the MK test should show increased evidence of positive selection in these regions relative to the rest of the genome. Simmons *et al.* (2008) used the MK test to determine that distinct strains of *Leptospirillum* group II detected within population genomic assemblies do not appear to be positively selected for adaptive differences with the dominant population, indicating that the ecotype model was not applicable to the population. The availability of metagenomic datasets, in particular those that provide a deep sampling of one or more natural populations, is providing an opportunity to test previously proposed evolutionary models. Currently, however, both the methodology to perform population genetic analysis on these kinds of data as well as the number of appropriate datasets are limited. It is clear that a continued effort in this field will help us garner a higher-resolution understanding of the relative importance of different evolutionary forces. One particular evolutionary force we have yet to discuss is the genetic change induced by the dynamic interplay between viruses and their hosts.

## The viral world and its role in perturbation and fine-tuning

In the majority of microbial ecosystems surveyed thus far, extracellular viral particles outnumber their archaeal, bacterial, and eukaryal hosts by at least one order of magnitude (Bergh *et al.*, 1989; Fuhrman, 1999). Overall, the Earth is a reservoir for an estimated 10^31^ viruses, most of which are bacteriophages (Breitbart & Rohwer, 2005). Viruses may be responsible for killing up to 25% of microbial cells per hour in the ocean (Heldal & Bratbak, 1991; Steward *et al.*, 1992), contributing to nutrient recycling. Thus, viruses have tremendous impacts on the Earth's biogeochemical cycles.

Viral diversity is significantly underrepresented in public sequence databases (Edwards & Rohwer, 2005). However, this is changing rapidly with the acquisition of extensive viral metagenomic sequences from multiple environments (Dinsdale *et al.*, 2008a). Apart from virus-focused studies that have revealed extensive viral genetic diversity (Breitbart *et al.*, 2002, 2003, 2004a, b; Angly *et al.*, 2006; Culley *et al.*, 2006; Zhang *et al.*, 2006; Schoenfeld *et al.*, 2008), several recent metagenomic studies have reported the simultaneous sampling of microorganisms and co-occurring viruses (DeLong *et al.*, 2006; Edwards *et al.*, 2006; Rusch *et al.*, 2007; Andersson & Banfield, 2008; Dinsdale *et al.*, 2008a; Williamson *et al.*, 2008). Such studies are providing the first glimpses into the dynamics of virus–host interactions. Furthermore, they suggest that such interactions may have a significant effect on fine-scale genetic heterogeneity within communities. In fact, viruses impact host genotypes in several ways: they mediate gene transfer between host populations, integrate into host genomes, and drive rapid diversification of host CRISPR loci involved in phage resistance.

Viruses reproduce in their host either by the lytic or by the lysogenic cycle. The lytic cycle is thought to be the dominant mode of virus proliferation, involving the destruction of the host cell through a burst event or the continuous secretion of viruses into the extracellular environment. In the lysogenic cycle, a temperate virus integrates its genome into the host's genome, becoming a provirus that can be transmitted to daughter cells until, at a later stage, it releases and the virus proliferates via the lytic cycle.

These two lifestyles allow viruses to be important mediators of genetic exchange in the environment (Ripp *et al.*, 1994; Jiang & Paul, 1998). As agents of gene transfer, viruses may supply the host with new genetic material in the form of integrated elements (reviewed by Faruque & Mekalanos, 2003; Sherwood, 2003; Brussow *et al.*, 2004) and replace cellular genes by viral nonorthologs (horizontal or lateral gene transfer; Filée *et al.*, 2002, 2003). In some cases, viruses are known to increase the short- and long-term survival fitness of the host (Brussow *et al.*, 2004). Cyanophages infecting *Synechococcus* and *Prochlorococcus* carry genes involved in photosynthesis (Mann *et al.*, 2003; Lindell *et al.*, 2004). The expression of cyanophage-encoded photosystem proteins (psbA/psbD) helps to support photosynthetic activity in the host during the infection cycle, providing photosynthetic gene-carrying cyanophages with a selective advantage (Lindell *et al.*, 2004). Viral *psbA* and *psbD* have been detected in open ocean metagenomic surveys (Venter *et al.*, 2004; Angly *et al.*, 2006; DeLong *et al.*, 2006; Rusch *et al.*, 2007). Sixty percent of *psbA* genes along the GOS sampling transect were of viral origin, suggesting that cyanophages may have a pronounced effect on global photosynthetic productivity (Sharon *et al.*, 2007). Moreover, phage *psbA* genes are evolving under levels of purifying selection that are virtually indistinguishable from those acting on host proteins (Zeidner *et al.*, 2005). Exchange and reshuffling of *psbA* genes occurs between *Synechococcus* and *Prochlorococcus* via phage intermediates, as well as between phages and hosts and between phages (Sullivan *et al.*, 2003). Consequently, cyanophages appear to play a role in both short- and long-term adaptation in host populations.

Little is known about the molecular mechanisms facilitating rapid genome evolution in microbial viruses. Comparative genomics suggest that the viral gene pool appears to be shaped primarily by illegitimate and homologous recombination (Hendrix, 2003; Martinsohn *et al.*, 2008). Apart from recombination, recently described diversity-generating retroelements (Liu *et al.*, 2002) allow viruses to generate adaptive diversity through a stochastic mechanism analogous to the mammalian immune system (Medhekar & Miller, 2007).

Recent evidence suggests that the viral gene pool extends across different biomes. Identical or near-identical bacteriophage-encoded genes have been identified in different ecosystems (Breitbart *et al.*, 2004a, b; Short & Suttle, 2005). Because of their similarity, these genes may have moved between environments within recent evolutionary history, for example within the last 1000–2000 years (Breitbart & Rohwer, 2005). Two distinct processes may explain the movement of bacteriophage-encoded genes from one biome to another:

1. *Transfer of single genetic elements*. Within natural virus populations, the rate of reassortment exceeds the rate of substitution (Silander *et al.*, 2005) and, hence, lateral gene transfer may be a mechanism for the global movement of viral genetic elements between biomes (Breitbart *et al.*, 2004a, b; Breitbart & Rohwer, 2005; Silander *et al.*, 2005).
2. *Immigration of phages*. Virus diversity in Yellowstone National Park hotsprings was primarily maintained by high rates of foreign immigration and recombination rather than mutation (Snyder *et al.*, 2007). Furthermore, transplanted viruses find hosts in foreign biomes (Sano *et al.*, 2004). These findings suggest that either identical microbial hosts are found in different environments or mobile viruses have broad host ranges (Jensen *et al.*, 1998; Sullivan *et al.*, 2003; Beumer & Robinson, 2005).

### Host defense mechanisms

Hosts and viruses are involved in a continuous evolutionary arms race. Archaeal and bacterial hosts have a number of viral defense mechanisms in their arsenal. These include restriction-modification systems (Wilson & Murray, 1991), cell-surface manipulations (Weitz *et al.*, 2005), exopolysaccharide production (Sutherland, 2001), biofilm formation (Sutherland *et al.*, 2004), abortive infection systems (Sturino & Klaenhammer, 2007) and the CRISPR system (recently reviewed by Sorek *et al.*, 2008). Pronounced variation in genomic regions related to these systems (exopolysaccharide synthesis cassettes and CRISPR loci) is apparent between strains of the same species, for example *Streptococcus thermophilus* (Bolotin *et al.*, 2004) and ‘*Candidatus* Accumulibacter phosphatis’ (Kunin *et al.*, 2008).

The CRISPR system has recently attracted considerable attention as it represents a putative archaeal and bacterial immune system for defense against foreign DNA (Makarova *et al.*, 2006). CRISPR genomic regions are comprised of a few to many tens (or even hundreds) of tandem-repeated DNA sequences, typically 21–47 bp in length, separated by nonrepetitive spacer sequences of approximately the same length and variable arrays of CRISPR-associated (*cas*) genes (Makarova *et al.*, 2006). Cas proteins share functional similarity with proteins involved in eukaryotic RNA interference systems and, hence, it has been hypothesized that spacers function analogously to small interfering RNAs (Makarova *et al.*, 2006). Although the exact functional mechanism of the CRISPR-system has yet to be determined, Barrangou *et al.* (2007) elegantly demonstrated in cultures of *S. thermophilus* that the CRISPR locus provides resistance against bacteriophages and that resistance specificity is determined by spacer-phage sequence similarity.

**Fig. 5:**
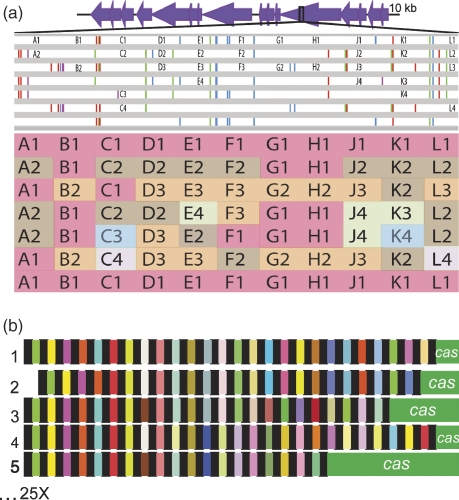
The dynamic interplay between viruses and their hosts (Andersson & Banfield, 2008). (a) Population structure of the AMDV2 virus population, showing extensive recombination between closely related sequence variants. Putative genes are displayed on top. Pattern of nucleotide polymorphisms (SNPs, colored bars) in a subset of sequencing reads within a region of the DNA polymerase gene. The region was divided into equally spaced blocks (A–L), and the alleles were numbered based on SNP patterns to the left of the label. In the summary table below, colors are assigned to alleles based on the read in which the allele first appears. (b) Schematic representation of the CRISPR locus of the corresponding host population, sampled 25 times, and characterized by an extensive diversity of spacer sequences (colored bars) in between the repeats (black bars). CRISPR loci grow unidirectionally, with a new spacer being introduced to the left of the neighboring CRISPR-associated protein machinery (*cas* genes). Because every cell is exposed to different viruses, the CRISPR spacer content, which reflects the natural history of the cell and its ancestry, might be unique for every single cell in the population.

More recently, Andersson & Banfield (2008) were able to use spacer sequences to retrieve corresponding viral sequences from community genomic datasets and assemble large viral genomic fragments. Using this targeted approach, virus–host dynamics were resolved by linkage of host-encoded spacer sequences to the corresponding viruses. CRISPRs are highly variable between closely related individuals and evolve rapidly (Tyson & Banfield, 2008). Only the most recently acquired spacers match coexisting viruses (Andersson & Banfield, 2008). This suggests that incorporation of new spacers into the CRISPR locus counteracts rapid local viral evolution and foreign immigration. Furthermore, visual analysis of viral contigs suggests that spacer evasion may occur predominantly through recombination (Fig. 5a). Consequently, viruses and hosts are locked into a continuous ‘arms race’ between the host's defenses and the virus counterdefenses, as symbolized by the Red Queen Principle (Van Valen, 1973).

### Models for viral population dynamics

Direct extrapolation from metagenomic data suggests that there may be *c*. 100 million distinct viral genotypes (Rohwer, 2003). This diversity is not partitioned equally across spatial scales, however, due to the fact that viruses (or at least some of their genes) move between biomes. The observation that viruses can be globally distributed but have high local diversity led to the development of the ‘Bank Model’ (Breitbart & Rohwer, 2005). This model assumes that only the most abundant viruses in a given environment are active, with the remaining low-abundance fraction being analogous to an inactive seed bank. Furthermore, only abundant viruses behave according to the ‘Kill-the-Winner’ hypothesis (Thingstad & Lignell, 1997), in which the dominant host population is reduced by viral attack, allowing a new host population to rise in frequency. The model is supported by rank-abundance curves indicating that the vast majority of viral genotypes are extremely rare (Breitbart & Rohwer, 2005).

Numerous findings indicate that the Bank Model may not accurately describe viral population dynamics in all/some environments. Significant lower host–virus ratios in extreme environments as well as short half-lives (48 h) in the marine environment are an indication that free-living viruses generally degrade rapidly (Wommack & Colwell, 2000; Breitbart *et al.*, 2004a, b), making a large bank of low-abundance and inactive extracellular lysing viruses unlikely. The model also does not account for the dynamics of nonlysing viruses, which are secreted from host cells without killing them [as in the hyperthermophilic archaeon *Sulfolobus tengchongensis* (Xiang *et al.*, 2005)]. Viral secretion is highly advantageous if fecundity is only slightly compromised relative to lytic bursts (Bull *et al.*, 2004). However, the abundance of nonlysing viruses in nature is not known, likely due to their inability to form plaques in plate count assays (representing a possible second ‘plate count anomaly’ in microbial ecology). These viruses would be classified as ‘inactive’ under the Breitbart–Rohwer Bank Model, but may in fact replicate slowly and continuously. The prevalence of nonlysing viruses may allow different viral genotypes to coinfect a microbial cell, resulting in extensive recombination within the cell.

Although simple ‘Kill-the-Winner’ scenarios are common in the laboratory environment, few studies suggest that this succession pattern is prevalent in natural communities (Mühling *et al.*, 2005; Martinez *et al.*, 2007). Detailed analysis of CRISPR spacers and coexisting viruses in AMD biofilms (Andersson & Banfield, 2008) suggests that the prolonged coevolution of virus–host pairs leads to broad genetic diversity within the local viral gene pool (Fig. 5a). Only one virus–host pair out of five virus populations analyzed in detail in AMD exhibited a pattern suggestive of recent virus immigration and a targeted selective sweep as predicted by the Bank Model. The observed heterogeneity among incorporated CRISPR spacers within microbial populations (Tyson & Banfield, 2008) suggests that host cells likely differ in their susceptibility to certain viruses. Concomitantly, due to the extensive variability among viral genotypes, viruses likely differ in their virulence. Thus, relatively stable coexisting host and virus populations seem possible (Andersson & Banfield, 2008). Only in a limited number of cases does a potent lysing virus emerge locally or immigrate from the Bank that results in a selective sweep among a dominant group of organisms. Consequently, in at least some environments, ‘Kill-the-Winner’ scenarios may be more the exception than the norm.

The patterns of spacer diversity within CRISPR loci suggest that virus population dynamics may be quite subtle. Bioinformatic and experimental evidence both indicate that novel spacers are added to only one end of the CRISPR locus nearest the *cas* genes, and that infection by novel viral types results in spacer addition (Barrangou *et al.*, 2007). Analysis of deeply sampled CRISPR loci in natural populations are consistent with this observation; spacers at one end of the locus are nearly identical in all individuals sampled, while at the opposite end each individual cell has a unique spacer complement (Andersson & Banfield, 2008; Tyson & Banfield, 2008;Fig. 5b). Hence, the evolution of the CRISPR spacer complement may be explained by the following scenario: infection by a novel viral genotype results in the lysis or weakening of most individuals, except those that are able to capture and incorporate a corresponding spacer into their CRISPR locus. At present, we do not know the fraction of individuals within a population that gain resistance by spacer addition, nor the rate at which viruses can evade CRISPR-acquired resistance via mutation or recombination. Resistant individuals would rapidly gain a selective advantage, leading to the fixation of the resistant spacer and its associated spacer inventory within the CRISPR locus. Under a straightforward ‘Kill-the-Winner’ scenario, we might expect this rapid rise of a single resistant host type to result in homogenization of the entire locus in the population, which appears inconsistent with virus population genomic data on hand at this time. However, if we assume that cells resistant to a certain viral genotype are being continually infected by mutated variants of the same virus or other viruses during their rise in frequency, diverse new spacers could be added to one end of the CRISPR locus while it is homogenized by selection on the other.

The observed heterogeneity in microbial hosts' spacer complements as well as the extensive viral genotypic diversity suggests that fine-scale variation is a major factor influencing host–virus dynamics. Future studies based on in-depth sampling of CRISPR spacers and corresponding viruses will determine the temporal and spatial scales important for virus–host evolution, and will result in more comprehensive models for virus–host dynamics.

## Conclusion

Community genomics is one among a diverse set of tools that can be applied to gain a greater understanding of microbial communities. The complexities revealed by these large and detailed datasets challenge us to consider a number of important new questions. Gene-centric analyses, as discussed above, allow construction of functional scaffolds to model metabolic interactions within a community (e.g. Warnecke *et al.*, 2007) as well as the determination of large-scale differences between the gene complements of distinct ecosystems (e.g. Tringe *et al.*, 2005; DeLong *et al.*, 2006; Dinsdale *et al.*, 2008a). It is now clear, however, that genetic variation within microbial communities is extensive at multiple levels. A gene-centric approach, while informative for certain questions, leaves this variation largely untouched. Community genomic data can provide significant insights into ecological and evolutionary dynamics within communities. This level of analysis is vital to a complete understanding of the form, function, and dynamics of variation within microbial consortia.

Our current understanding of the role of within-population genetic heterogeneity is limited. Theoretical models suggest that some fraction of this variation could result from neutral evolutionary processes such as mutation, recombination, and genetic drift, while others have suggested that sequence variation demonstrates niche-specific adaptation. The wider application of established population genetic tools to detect signatures of selection in community genomic sequence data could shed significant light on this question. To date, experimental data on the expression of genes in hypervariable regions suggest that at least some genotypic diversity contributes to community functioning. Because of the limited number of studies that have addressed the relevance of fine-scale variation in natural populations, it is premature to make any general conclusions regarding its fitness effects.

The importance of fine-scale genetic variation within microbial populations is an interesting question from a basic scientific perspective, but it also has important practical implications. Human society relies heavily on microorganisms. Over the millennia, humans have learnt to harness and engineer several microbial processes. These range from food preservation (Ross *et al.*, 2002) to the treatment of waste (Daims *et al.*, 2006) to the provision of raw materials for manufacturing (Bosecker, 1997). To return to our initial orchestra analogy, although we are attempting to take over the role of the microbial community conductor, we have limited knowledge of the score and how it is played. Metagenomics, in combination with functional approaches, offers opportunities to help improve our performance. Improvement is necessary, because our current lack of understanding often results in mediocre process performances and intermittent failures.

Particularly problematic are phage attacks that represent a major financial burden to the fermentation industry (Petty *et al.*, 2007). In order to improve the operational stability of such microbial processes, a detailed understanding of community dynamics is essential. In particular, the elucidation of virus–host interactions in relation to the recent discovery of the CRISPR system holds great promise for future biotechnological applications. This knowledge might allow us to use the CRISPR system to engineer microbial communities. For example, the system could be used to shape community composition either by improving resistance to phage predation or by silencing specific genes within microbial populations. Moreover, in the light of current challenges imposed by antibiotic resistance (Kluytmans-VandenBergh & Kluytmans, 2006), detailed knowledge of virus–host interactions deduced from studying CRISPR spacers and their targeted viruses might lead to novel infection treatment technologies. For example, rapid CRISPR spacer typing of pathogenic bacteria may provide the foundation for synthetic phage therapy, which could be facilitated by current advances in the field of synthetic biology.

It is important that biotechnology, including the emerging field of synthetic biology, reflects on the lessons learned from failed attempts to use clonal isolates for the engineering of microbial systems, for example bioaugmentation (El Fantroussi & Agathos, 2005). Furthermore, considering the extensive population-level heterogeneity, it could be fruitful to revisit the current quest for the ideal biocatalyst (Burton *et al.*, 2002), using strategies that exploit the diversity in natural communities. It is interesting to note that the most widely applied and one of the most successful ‘bio-catalysts’, activated-sludge in wastewater treatment, harnesses natural communities with their inherent population-level heterogeneity (García-Martín *et al.*, 2006; Kunin *et al.*, 2008; Wilmes *et al.*, 2008a). The question now is whether this heterogeneity confers system resilience and whether communities can be engineered to provide certain services more efficiently? To quote Leonardo Da Vinci: ‘Human subtlety will never devise an invention more beautiful, more simple or more direct than does Nature, because in her inventions, nothing is lacking and nothing is superfluous.’

## Statement

Re-use of this article is permitted in accordance with the Creative Commons Deed, Attribution 2.5, which does not permit commercial exploitation.
